# Efficacy of Two Laser Treatment Strategies for Breast Cancer Survivors With Genitourinary Syndrome of Menopause

**DOI:** 10.7759/cureus.38604

**Published:** 2023-05-05

**Authors:** Nobuo Okui, Machiko Okui, Yuko Kouno, Kaori Nakano, Marco Gambacciani

**Affiliations:** 1 Department of Dentistry, Kanagawa Dental University, Kanagawa, JPN; 2 Department of Urology, Dr. Okui’s Urogynecology and Urology Clinic, Kanagawa, JPN; 3 Menopause and Osteoporosis Unit, San Rossore Clinical Center, Pisa, ITA

**Keywords:** dyspareunia, vulvodynia, propensity score matching, superficial vulvar pain, postmenopausal women, laser treatment, genitourinary syndrome of menopause

## Abstract

Background

A typical symptom of patients with genitourinary syndrome of menopause (GSM) is dyspareunia. Dyspareunia has been thought to be caused by vaginal dryness. In recent years, a survey of breast cancer survivors (BCS) with GSM has shown that para-hymen is the most painful. Dyspareunia and superficial vulvar pain (vulvodynia) may be closely linked. A recent study showed that vulvodynia is very common in BCS. Therefore, we believe treatment targeting the vagina and the vulva is necessary for pain in BCS with GSM. We hypothesized that treating both the vagina and the vulva would solve the problem of BCS with GSM. We compared the vaginal erbium SMOOTH mode laser (VEL) and neodymium-doped yttrium-aluminum-garnet (Nd:YAG) laser (VEL+Nd:YAG) combination treatment over time. This study explores therapeutic targets for pain in BCS with GSM.

Methodology

This retrospective, case-control study targeted sexually active BCS who reported GSM with vulvodynia and dyspareunia. After all women enrolled in the VEL treatment group had completed treatment, we treated women enrolled in the VEL+Nd:YAG treatment group. A total of 256 women who received either VEL+Nd:YAG or VEL were enrolled. Propensity score (PS)-matching analysis was used to compare two-year postoperative data retrospectively. The PS-matching results registered 102 patients in the VEL+Nd:YAG group and 102 patients in the VEL group. Symptoms were assessed using the visual analog scale (VAS) for vulvodynia before and after laser treatment for one, three, six, 12, and 24 months after completion. As a preliminary study, the vulvodynia swab test confirmed the causative location of dyspareunia. Moreover, the Female Sexual Function Index (FSFI) and Vaginal Health Index Score (VHIS) were assessed. FSFI and VHIS were treated as supplement research because the conditions were unmet.

Results

In the vulvodynia swab test, dyspareunia, and para-hymen (especially at 4 o’clock and 9 o’clock), all felt pain, and only a few felt pain in the vagina and labia. FSFI improved significantly in the VEL+Nd:YAG group and persisted for two years. VHIS improved equally in both groups and was not significantly different. After the first laser application, the VEL+Nd:YAG and the VEL groups showed sustained efficacy and safety in vulvodynia. Baseline VAS scores (8.74 ± 0.72 vs. 8.79 ± 0.74; p = 0.564) were similar in both groups. Both groups had a significant (p < 0.001) decrease in the VAS score. The VAS values in the VEL+Nd:YAG group and the VEL group decreased from the pretreatment to 3.79 ± 0.63 (p < 0.001 vs. baseline) and 5.56 ± 0.89 (p < 0.001 vs. baseline) after the third treatments, respectively. After 24 months, the VAS value in the VEL+Nd:YAG group and the VEL group was at 4.43 ± 1.38 (p < 0.001 vs. baseline) and 5.56 ± 0.89 (p < 0.001 vs. baseline), respectively. The side effects in both groups were short-term and minor.

Conclusions

Both VEL+NdYAG and VEL effectively and safely treat GSM dyspareunia and vulvodynia in BCS. Comparing the two groups, we confirmed that VEL+Nd:YAG treatment of the vaginal vestibule and vaginal opening reduced superficial vulvar pain more effectively, extensively, and over a longer period than VEL. The results of the vulvodynia swab test, FSFI, and VHIS suggest that the vulva and the vagina are important therapeutic targets for pain in BCS with GSM. The importance of treating the vulvar area for superficial pain and dyspareunia in GSM has been emphasized.

## Introduction

A common symptom of genitourinary syndrome of menopause (GSM) is dyspareunia due to vulvovaginal atrophy (VVA), which refers to pain during intercourse. Moreover, patients will experience pain in various situations in their daily life. Such pain problems are often associated with decreased quality of life (QoL) in postmenopausal women (PMW) with GSM [1,2]. Recently, a cohort study showed that the anatomical location of pain in moderate/severe dyspareunia due to GSM is 98% common in the vulvar vestibule (just outside the hymen) [3]. This study tested 55 eligible women (mean age was 59.5 ± 6.8 years, and the duration of dyspareunia was 6.2 ± 4.3 years). The mean dyspareunia score was 7.3 ± 1.8 using the Numerical Rating Scale (0-10). The most often described symptoms are burning and raw. Median pain scores from swab touch just outside the hymen vulvar vestibule ranged from 4 to 5/10 [3]. For sexually active women, this pain is described using the term dyspareunia [3]. In patients with unexplained pain in the vulva that has persisted for more than three months, this pain has come to be termed vulvodynia [4]. Vulvodynia includes many pathologies, not only GSM. It has been implicated in the interaction of central and peripheral pain mechanisms, pelvic floor muscle and autonomic dysfunction, depression, anxiety, childhood abuse, and cognitive, behavioral, and interpersonal factors [4]. Murina et al. conducted a case-control study of vestibulodynia (VBD), a form of vulvodynia, comparing a group of women with VBD, a group of women with GSM, and a group of healthy women [5]. In this study, labial ultrasound examined the thickness of the vestibular mucosa. VBD and GSM groups were found to have thinner vestibular mucosa compared to age-matched healthy women. This fact was significantly correlated with burning/pain intensity and dyspareunia severity. Therefore, studying GSM concerning vulvodynia is worthwhile [5].

Previously, vulvodynia was considered a rare disease; however, recent studies have shown that it affects more people. In particular, a previous study reported a higher prevalence among breast cancer survivors (BCS) [6]. A study of 100 postmenopausal patients with a history of breast or endometrial cancer showed that vestibular irritation in 55%, vulvar atrophy in 66% (mild 50%, moderate 12%, severe 4%), vulvar irritation in 54% (mild 26%, moderate 20%, severe 7%), and vaginal irritation in 36% (mild 23%, moderate 9%, severe 4%) of patients [6]. From this previous study, we believe both the vagina and the vulva should be explored and framed in treatment approaches for women with BCS.

GSM treatments include hormonal and non-hormonal therapies [7,8]. Severe GSM in BCS is often caused by anti-estrogen therapy [9,10]. Female hormone use, including low-dose topical estrogen administration, is contraindicated in these patients [11]. Current practice treatments include lifestyle changes and non-hormonal therapies (vaginal moisturizers, lubricants, gels). However, if these are not effective in relieving symptoms, other options such as ospemifene, topical androgens, and intravaginal dehydroepiandrosterone (Prasterone) may be considered [12].

Laser therapies (erbium or CO_2_ lasers) are commonly employed as well. Current data suggest that laser therapy is effective for VVA in BCS. However, safety remains controversial, and there are undeniable major concerns with all of these treatments [12]. Non-ablative erbium:yttrium-aluminum-garnet (Er:YAG) vaginal laser is attracting attention in terms of both safety and efficacy.

In recent years, the 2,940 nm non-ablative Er:YAG vaginal laser (vaginal erbium SMOOTH mode laser [VEL]) has been reported to improve GSM symptoms of PMW and BCS [13-16]. However, dyspareunia has not been shown to respond entirely to any energy-based device (EBD) treatment [17]. In addition, women with a history of breast cancer were found to experience the same level of recurring pain present before treatment, even after two years of VEL treatment [15].

In 2022, a pilot erbium-doped and neodymium-doped laser combination (VEL+Nd:YAG) therapy study, which combined the VEL protocol with neodymium-doped YAG (Nd:YAG) laser treatment for the posterior commissure and vulva, was conducted on women with GSM [18]. The Nd:YAG laser is a 1,064 nm crystal laser that invokes deeper light into human tissue. With its long pulse and duration in seconds (PIANO mode), this laser is appropriate for uniform bulk heating of subcutaneous fat and dermis. Long-pulse PIANO mode durations can gently extend deeper structures for only a few seconds [18]. This pilot study showed that VEL+Nd:YAG can improve superficial vulvar pain more effectively than VEL alone [18]. Another study reported that VEL+Nd:YAG was effective for severe vulvar pain associated with interstitial cystitis [19]. The PIANO mode has been popular for several years, and the VEL+Nd:YAG has already been implemented in many medical protocols. The long-term results have been confirmed in a retrospective study [18,19].

The present study reports a retrospective analysis of the long-term outcomes of VEL and VEL+Nd:YAG using a propensity score (PS)-matching analysis in BCS treated for superficial vulvar pain.

## Materials and methods

Patient selection criteria

We recruited BCS women with GSM using internet web service advertisements from 2016. On our recruitment website, we explained GSM and breast cancer. For our recruitment website, advertisements were displayed when searching for “breast cancer,” “dyspareunia,” “vulva pain,” “labial pain,” and “vaginal pain” in Google search. We made it possible for patients to make reservations for our clinic’s “GSM-dedicated office” on the Google platform from the recruitment website.

Among the women diagnosed with GSM [20] following breast cancer treatment at our Department’s “GSM-dedicated office” and the Kanagawa Dental University between January 2016 and December 2020, we included those who met the inclusion and exclusion criteria and had undergone laser treatment with either VEL+Nd:YAG or VEL. We interviewed patients verbally about their sexual activity.

The inclusion criteria for laser treatment were the presence of superficial vulvar pain (dyspareunia) for three months or longer in sexually active women, negative Pap smear, and hormonal profile (estradiol at <25 pg/mL and follicle-stimulating hormone at ≥40 U/L). Vulvodynia was defined as pain in the vulva lasting three months or more and not caused by any other medical condition such as infection, skin disease, cancer, or stones.

The exclusion criteria included the (1) use of lubrication, topical preparations, hormones, or other medications for menopausal symptoms during the three months before treatment; (2) presence of urinary or genital lesions, scars, or infections; (3) vaginal/uterine abnormal bleeding; (4) use of photosensitizers or a history of photosensitivity; (5) pelvic organ prolapse (Classification Pelvic Organ Prolapse Quantification System (POPQ) grades II-III); (6) severe mental illness that may interfere with the examination; and (7) chronic or severe diseases that may interfere with treatment.

The recruiting website had an average of 1 million views per month. The average number of clicks per month was 18,200. Women who browsed the recruitment website visited our clinic seeking treatment for GSM and similar conditions. A total of 4,035 women visited our clinic. In total, 2,015 women were diagnosed with GSM. There were 282 BCS. Of these, 266 patients gave written consent for our study. Our clinic introduced VEL treatment equipment in 2016 and VEL+Nd:YAG treatment equipment in 2018. All individuals enrolled after 2016 underwent VEL treatment. All individuals enrolled after 2018 underwent VEL+Nd:YAG treatment. We decided to conduct a retrospective study in 2022 when all patients had completed at least two years of treatment. Ten patients withdrew their consent during the opt-out period.

Our retrospective study started, and we researched these patients in 2022. Our retrospective investigation included 256 women who met the inclusion and exclusion criteria and had undergone laser treatment. The VEL+Nd:YAG was used in 137 women, and VEL alone was used in 119 women. We excluded patients (16 women from the VEL+Nd:YAG group and six from the VEL group) with less than two years of follow-up and those with missing data. Finally, 234 women were included in the study (VEL+Nd:YAG = 121 women; VEL = 113 women). The sample sizes of the two groups were estimated to provide 95% confidence intervals (CIs) with 5% errors.

**Figure 1:**
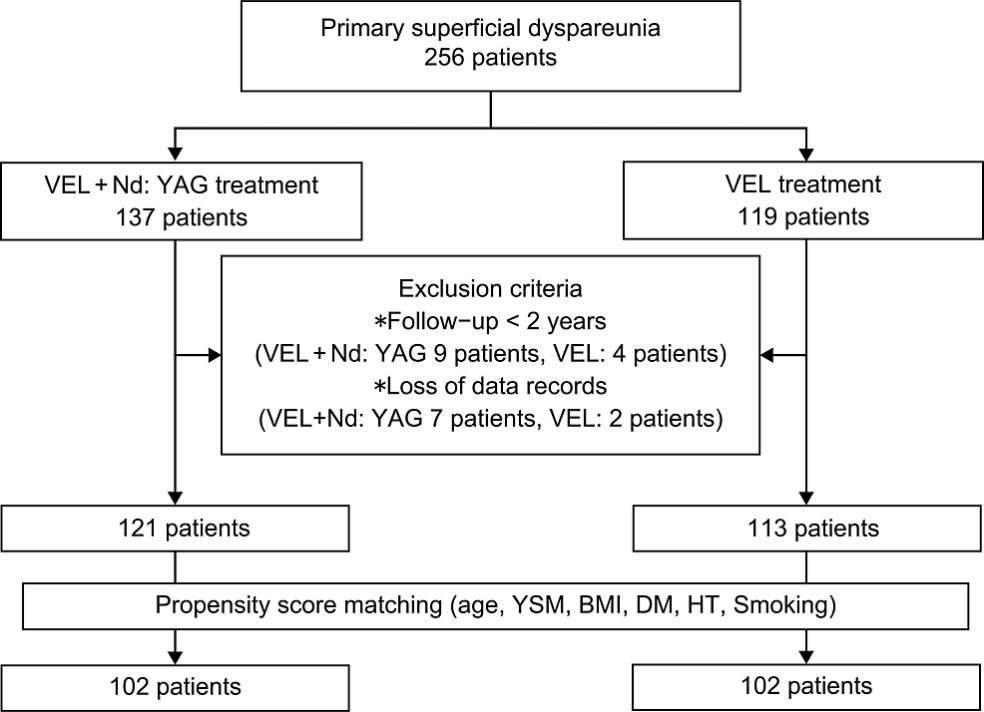
Study flowchart showing the inclusion and exclusion criteria. VEL: vaginal erbium SMOOTH mode laser treatment; VEL+Nd:YAG: VEL and neodymium-doped yttrium-aluminum-garnet (Nd:YAG) laser combination treatment; YSM: years since menopause; BMI: body mass index; DM: diabetes mellitus; HT: hypertension

We conducted a PS-matching analysis that used age at surgery, age at menopause, years since menopause (YSM), body mass index (BMI), diabetes mellitus (DM), hypertension (HT), and smoking as confounding factors. Subsequently, the participants were divided into two groups, namely, VEL+Nd:YAG and VEL (Figure 1).

Breast cancer stages and treatment history information

Breast cancer stage [21] and treatment history were entered online by the patient before visiting our clinic. At the first visit, we reviewed previous treatment records. Regarding the classification of breast cancer treatment, we mimicked previous studies [22] and created the classification.

Evaluation method

Women were treated with three laser sessions (L1, L2, and L3) every 30 days, with a screening visit two to four weeks before the first laser treatment (T0, baseline). Follow-up visits were performed at one (T1), three (T3), six (T6), 12 (T12), and 24 months (T24) after completion of the treatment. Patient eligibility was confirmed during the baseline visit, and age at treatment, age at menopause, YSM, BMI, DM, HT, and smoking status were then investigated. If DM and HT required medication, the patient was considered ill. Superficial vulvar pain was assessed using the visual analog scale (VAS) at each visit and before each laser application session (range = 0-10; 0 = a complete absence of symptoms, 10 = worst possible symptoms).

At T0, all patients underwent a doctor’s examination. VAS, vulvodynia swab tests, the Female Sexual Function Index (FSFI), and Vaginal Health Index Score (VHIS) were assessed.

**Figure 2:**
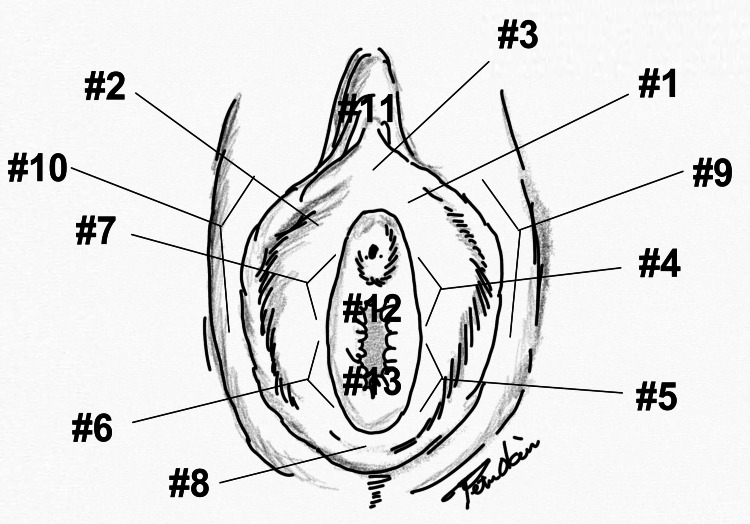
The vulvodynia swab test. The location of touch sites: 1:00 (#1), 11:00 (#2), 3~4:00 (#4), 5:00(#5), 6:00 (#8), 7~8:00 (#6), 9:00 (#7), 11:00 (#2), 12:00 (#3), clitoris (#11), right labia majora (#10), left labia majora (#9), anterior vaginal wall (#12), and posterior vaginal wall (#13). Artist: Peter Nobuo Okui.

The vulvodynia swab test is used to determine systemically or locally induced vulvodynia. Pressure is systematically applied to different parts of the vulva (vestibule, clitoris, etc.) to assess the degree and characteristics of pain to execute this test. For this study, the test was performed at the cardinal positions, namely, 1:00 (#1), 11:00 (#2), 3~4:00 (#4), 5:00(#5), 6:00 (#8), 7-8:00 (#6), 9:00 (#7), 11:00 (#2), 12:00 (#3), the clitoris (#11), right labia majora (#10), left labia majora (#9), anterior vaginal wall (#12), and posterior vaginal wall (#13). Figure 2 shows where to apply tenderness. We mimicked previous studies [23].

We also checked the vaginal opening. For positions where the pain was reported, the patient was asked to quantify the pain while referencing a VAS score from 0 (no pain) to 10 (severe pain). This test is vital in correlating pain indicated by the VAS with the localization of pain by the clinician.

The FSFI is a 19-item self-assessment questionnaire used to evaluate female sexual dysfunction (FDS) [24]. The questionnaire was used according to the patient’s language (English, Japanese, and Chinese). This questionnaire assesses six areas of sexual function, namely, desire, arousal, lubrication, orgasm, satisfaction, and pain in females. These questions are scored for sexual activity over the previous four weeks. The individual domain score is calculated by multiplying the individual domain questions with the domain factors. Adding the scores for each domain yields the total score. Scores vary from a minimum of 1.2 to a maximum of 36. Scores below 26.55 are classified as FSD [24]. The biggest concern is that Asian (mainly Japanese) women tend to underestimate their sexual function [25]. Moreover, it is known that the Japanese population has become less sexually active. According to previous studies, the average total score of the sexual function index in women has changed from 14.6 in 2012 to 12.5 in 2019 (p < 0.001). The percentage of women who “have no sexual intercourse” with their partners is rising [25]. We treated the FSFI as a supplementary study in our long-term observational study.

The VHIS, which is a tool for objectively diagnosing vaginal health, involves evaluating the five items of overall elasticity, fluid secretion characteristics, vaginal pH range, epithelial mucosa, and moisture on a scale of 1 (none), 2 (poor), 3 (fair), 4 (good), and 5 (excellent). A total VHIS score of <15 indicates poor vaginal health [24]. Studies with VHIS require separate consultations by two physicians who do not know the details. In this study, doctors knew which group the patients belonged to. For this reason, we chose to report VHIS as a supplement study.

Treatment procedure

Patients received laser treatment without preparation, anesthesia, or post-treatment medication at the day surgery center. In each patient, the perineum and vagina were cleaned.

The treatment procedure for the VEL+Nd:YAG group consisted of two steps. In the first (VEL) step, renovalase intravaginal protocol (SP Dynamis laser device, Fotona, Slovenia) was applied using a glass speculum and R11-GC laser probe in smooth mode at 7 mm spot, 1.75 J/cm^2^, and 1.6 Hz. The laser energy was delivered circularly, and each pulse covered 360° of the vaginal canal. Seven laser pulses were emitted every 5 mm along the vaginal canal, and this irradiation was repeated three times [19]. In the second step, an Nd:YAG laser (from the same laser system) irradiated the posterior labial commissure. The Nd:YAG laser energy was delivered with an R33 laser probe in PIANO mode at a 9 mm spot, 90 J/cm^2^, and 5.0 s pulse duration. The treated area received six passes with a laser beam in the brushing (painting) mode [18,19]. Patients in the VEL group were treated only with the renovalase intravaginal protocol. There were no special postoperative care steps, except women were advised to avoid intercourse for at least a week after each treatment session.

Treatment selection

From 2016, all women were treated with VEL alone; from 2018, all were treated with VEL+Nd:YAG. Both treatments cost the same. Therefore, the cost was not a differentiating factor between the two groups regarding women’s evaluation of treatment efficacy. Both treatments were not covered by government insurance.

Statistical analyses

Continuous variables were expressed as mean ± standard deviation (mean ± SD). Student’s t-test was used to compare continuous variables between the two groups, and the chi-square test was used to compare the incidence of each parameter. Pearson’s correlation coefficient was used to examine the relationship between VAS and the vulvodynia swab test. Statistical significance was set at p ≤ 0.05.

The difference between T0 and T3 (Δ_T0/T23_) and T0 and T24 (Δ_T0/T24_) were calculated and compared between the two groups. Multiple linear regression models examined how women’s characteristics (YSM and age) and treatment modality affected the Δ_T0/T24_. Variables in the regression model were selected based on the hypotheses and biological specificity of the dependent variable.

All analyses were performed using the statistical software R version 2.15.1 (R Core Team, Vienna, Austria) and the EZR package on the Windows 10 version of the 1903 operating system (Microsoft Corp.).

## Results

The baseline demographics of the enrolled women are shown in Figure 1 and Table 1. The PS-matching results included 102 patients each in the VEL+Nd:YAG and the VEL groups. All women were Asians (160 Japanese, 16 Chinese, 18 Korean, five Filipino, one Vietnamese, one Sri Lankan, and one Indian).

**Table 1:** Baseline demographics of participating women after propensity score matching. Each date shows average ± standard deviation. *: Significant difference between the two groups. YSM: years since menopause (years); BMI: body mass index (kg/m^2^); DM: diabetes mellitus; HT: hypertension; VEL: vaginal erbium SMOOTH mode laser treatment; VEL+Nd:YAG: VEL and neodymium-doped yttrium-aluminum-garnet laser combination treatment

	VEL+Nd:YAG group	VEL group	P-value*
Number of women	102	102	
Age (years)	59.83 ± 1.41	59.94 ± 1.41	0.586
Age at menopause (years)	48.83 ± 2.01	49.00 ± 1.86	0.539
YSM	11.00 ± 1.40	10.94 ± 1.39	0.764
BMI	22.91 ± 2.51	22.68 ± 2.74	0.523
The proportion of DM women (%)	8 ± 7.8	7 ± 6.9	1.00
The proportion of HT women (%)	10 ± 9.8	11 ± 10.8	1.00
The proportion of smokers (%)	5 ± 4.9	6 ± 5.9	1.00
Follow-up period (years)	2.50 ± 0.51	2.53 ± 0.54	0.750

No significant differences were observed in age at treatment, age at menopause, YSM, BMI, and rates of various diseases (DM, HT, smokers) at the baseline (T0) between the two groups (Table 1).

Table 2 shows the breast cancer stage and treatment course at T0. There was no significant difference in breast cancer stage between the two groups after PS matching.

**Table 2:** Characteristics of breast cancer treatment after propensity score matching. *: Significant difference between the two groups. VEL: vaginal erbium SMOOTH mode laser treatment; VEL+Nd:YAG: VEL and neodymium-doped yttrium-aluminum-garnet laser combination treatment; AIs: aromatase inhibitors

	VEL+Nd:YAG group	VEL group	P-value*
Number of women	102	102	
Stage of breast cancer			
Stage I	51%	58%	0.328
Stage II	31%	30%	0.880
Stage III	5%	8%	0.392
Stage IV	2%	0%	0.157
Stage unknown	11%	4%	0.037
Chemotherapy			
No chemotherapy	66%	64%	0.768
Chemotherapy	34%	36%	0.560
Tamoxifen use			
No tamoxifen	23%	32%	0.156
0–2 years	28%	27%	0.875
2–5 years	20%	33%	0.037*
5 + years	29%	8%	<0.001*
AIs use			
No AI	47%	34%	0.062
0–2 years	14%	27%	0.023*
2–5 years	34%	31%	0.653
5 + years	29%	8%	<0.001*

There was a difference between the two groups regarding the number of patients who used tamoxifen for more than two years as treatment. There was also a significant difference between the two groups regarding the number of patients who had used aromatase inhibitors. Tamoxifen users were more in the VEL group, and aromatase inhibitors users were more in the VEL+Nd:YAG group (Table 2).

The location of the pain

Table 3 shows the correlation between vulvodynia swab tests and the VAS. We pooled the number of women who had positive swab tests. The most painful one was para-hymen; most patients felt pain in #4. Pearson’s correlation showed the relationship between VAS and the swab test (#4; strongly correlated, #3, 5, 6, and 7; correlated). Provoked pain in the vaginal wall was 2.5% in the anterior and 1.28% in the posterior walls. Notably, 71% (184/256) of the patients had unprovoked pain in the vulvar vestibule. It corresponded to the location of the strongest pain induced by the swab test.

**Table 3:** Correlation between vulvodynia swab tests and Visual Analog Scale score. The location of touch sites: 1:00 (#1), 11:00 (#2), 3~4:00 (#4), 5:00(#5), 6:00 (#8), 7~8:00 (#6), 9:00 (#7), 11:00 (#2), 12:00 (#3), clitoris (#11), right labia majora (#10), left labia majora (#9), anterior vaginal wall (#12), and posterior vaginal wall (#13). ***: Strong positive coefficient; **: moderate positive coefficient; *: mild positive coefficient

Location	Positive women (%)	Correlation coefficient
Anterior		
#1	21.7%	0.08
#2	17.1%	0.08
Para-hymen		
#3	78.2%	0.15*
#4	98%	0.47***
#5	85%	0.24*
#6	79.9%	0.23*
#7	89.7%	0.24*
#8	23.9%	0.016
Labia		
#9	11.6%	0.031
#10	10.3%	0.03
Clitoris		
#11	21.8%	0.013
Vaginal wall		
#12	2.5%	0.10
#13	1.28%	0.058

Effects of laser therapy over time

Table 4 shows the VAS scores of both groups for superficial vulvar pain over time. Baseline VAS scores (T0) (8.74 ± 0.72 vs. 8.79 ± 0.74; p = 0.564) were similar in both groups. Both groups had a significant (p < 0.001) decrease in the VAS score.

**Table 4:** Postoperative outcomes in the Visual Analog Scale score. L1: The first laser treatment; L2: the second laser treatment; L3: the third laser treatment. T0: A screening visit two to four weeks before the first laser treatment (baseline); T1: follow-up visits one month after L3; T3: follow-up visits three months after L3; T6: follow-up visits six months after L3; T12: follow-up visits 12 months after L3; T24: follow-up visits 24 months after L3. Each date shows average ± standard deviation. *: Significant difference between the two groups. VEL: vaginal erbium SMOOTH mode laser treatment; VEL+Nd:YAG: VEL and neodymium-doped yttrium-aluminum-garnet laser combination treatment

	VEL+Nd:YAG group	VEL group	P-value*
T0	8.74 ± 0.72	8.79 ± 0.74	0.564
L1	4.54 ± 1.04	7.07 ± 0.35	<0.001
L2	4.77 ± 0.73	6.03 ± 0.76	<0.001
L3	3.79 ± 0.63	5.56 ± 0.89	<0.001
T1	3.10 ± 0.81	5.26 ± 0.81	<0.001
T3	3.03 ± 0.90	5.71 ± 0.82	<0.001
T6	2.93 ± 0.95	5.73 ± 0.82	<0.001
T12	3.81 ± 1.11	6.93 ± 0.93	<0.001
T24	4.43 ± 1.38	8.44 ± 1.09	<0.001

The VAS values ​​in the VEL+Nd:YAG group decreased from the pretreatment 8.74 ± 0.72 to 4.54 ± 1.04, 4.77 ± 0.73, and 3.79 ± 0.63 (p < 0.001 vs. baseline) after the first, second, and third treatments, respectively. After 24 months, the VAS value was 4.43 ± 1.38 (p < 0.001 vs. baseline).

VAS values ​​in the VEL group decreased from the pretreatment 8.79 ± 0.74 to 7.07 ± 0.35, 6.03 ± 0.76, and 5.56 ± 0.89 after the first, second, and third treatments, respectively (p < 0.001 vs. baseline). After 24 months, the VAS value was 8.44 ± 1.09 (p < 0.001 vs. baseline).

A significant reduction in the VAS scores was noted in both groups after the first laser treatment. Furthermore, the VAS scores were significantly lower in the VEL+Nd:YAG group at all follow-ups.

The therapeutic change between the two treatment groups, calculated as Δ_T0/T3_, was significantly different between the two groups (-5.71 ± 1.04 points ​​in the VEL+Nd:YAG group vs. -3.09 ± 0.88 points in the VEL group; p < 0.001). ​​In the VEL+Nd:YAG group, Δ_T0/T24_ showed a sustained therapeutic effect (-4.30 ± 1.44 points), whereas, in the VEL group, ΔT0/T24 (- 0.35 ± 0.93 points) showed a recurrence of superficial vulvar pain (between groups; p < 0.001).

For Δ_T0/T24_, a multiple linear regression model that considered the age at treatment, YSM, and treatment as variables, we had an R^2^ marginal of 0.7526 and an R^2^ conditional of 0.7476. Age at treatment (estimate = -0.10; 95% CI = -0.22 to 0.012; p = 0.081) and YSM (estimate = -0.078; 95% CI = -0.20 - 0.04; p = 0.19) did not affect Δ_T0/T24_. Only the type of laser treatment performed significantly influenced the outcome (estimate = 3.97; 95% CI = 3.65 - -4.29; p < 0.001).

Complications

No superficial surgical site infections (SSIs) were observed. The most frequent complaints among the participants were transient moderate-heating sensations during treatment (VEL+Nd:YAG group = one vs. VEL group = one; p = 1.00), mild pain (VEL+Nd:YAG group = one vs. VEL group = two; p = 0.563), vaginal discharge (VEL+Nd:YAG group = four vs. VEL group = three; p = 0.702), mild edema (VEL+Nd:YAG group = one vs. VEL group = one; p = 1.00), dryness (VEL+Nd:YAG group = zero vs. VEL group = one; p = 0.319), transient de novo urinary incontinence (VEL+Nd:YAG group = one vs. VEL group = zero; p = 0.319), and itching (VEL+Nd:YAG group = zero vs. VEL group = one; p = 0.319). All minor complications lasted only a few days after treatment, and no participant complained of adverse events lasting longer than a year.

Supplement study

Table 5 shows changes in VHIS. At T0, the VEL+Nd:YAG group was 11.97 ± 2.85, and the VEL group was 11.88 ± 2.99. Neither had a healthy vagina. Both groups improved similarly with laser treatment. At T12, the VEL+Nd:YAG and VEL groups were 16.21 ± 3.42 and 16.59 ± 3.31, respectively. However, after 24 months, the vagina returned to its original state. The two groups were 14.92 ± 4.71 and 14.92 ± 3.86, respectively. There was no significant difference between the two groups.

**Table 5:** Changes in scores of VHIS. Table each date shows average ± standard deviation. T0: A screening visit two to four weeks before the first laser treatment ± baseline); T12: follow-up visits 12 months after the third laser treatment ± L3); T24: follow-up visits 24 months after L3. VEL: vaginal erbium SMOOTH mode laser treatment; VEL+Nd:YAG: VEL and neodymium-doped yttrium-aluminum-garnet laser combination treatment; VHIS: Vaginal Health Index Score

	VEL+Nd:YAG group	VEL group	P-value*
Number of women	102	102	
T0			
VHIS	11.97 ± 2.85	11.88 ± 2.99	0.830
Overall elasticity	2.31 ± 0.64	2.29 ± 0.68	0.833
Fluid secretion characteristics	2.56 ± 0.67	2.58 ± 0.78	0.847
Vaginal pH range	2.56 ± 0.67	2.54 ± 0.73	0.841
Epithelial mucosa	2.56 ± 0.67	2.41 ± 0.71	0.129
Moisture	1.98 ± 0.42	2.06 ± 0.46	0.207
T12			
VHIS	16.21 ± 3.42	16.59 ± 3.31	0.418
Overall elasticity	3.47 ± 0.86	3.24 ± 0.75	0.039
Fluid secretion characteristics	3.67 ± 0.84	3.52 ± 0.83	0.209
Vaginal pH range	3.48 ± 0.7	3.67 ± 0.84	0.102
Epithelial mucosa	3.67 ± 0.84	3.35 ± 0.75	0.0051
Moisture	3.67 ± 0.84	3.52 ± 0.83	0.209
T24			
VHIS	14.92 ± 4.71	14.92 ± 3.86	1.000
Overall elasticity	2.92 ± 1.00	2.90 ± 0.85	0.880
Fluid secretion characteristics	3.15 ± 1.01	3.19 ± 0.91	0.771
Vaginal pH range	3.15 ± 1.01	3.15 ± 0.87	1.000
Epithelial mucosa	3.15 ± 1.01	3.02 ± 0.87	0.102
Moisture	2.56 ± 0.83	2.67 ± 0.67	0.306

Table 6 shows changes in FSFI. This questionnaire asks about patients’ sex life. Eight women at T12 and another 10 women at T24 declined the questionnaire. The reason was the partner’s loss (divorce, bereavement) for seven women, and 11 women did not want to answer. PS matching was performed on 215 women who answered FSFI at three time points, namely, T0, T12, and T24. The VEL+Nd:YAG and the VEL groups each had 89 women. We focused on total FSFI. At T0, the VEL+Nd:YAG and VEL groups were 15.87 ± 4.69 and 15.57 ± 4.21, respectively. There was no significant difference. T12 yielded 20.89 ± 5.42 and 18.06 ± 5.86, respectively. The VEL+Nd:YAG group performed significantly better. For T24, they were 20.47 ± 5.97 and 15.88 ± 4.71, respectively. The significance of the VEL+Nd:YAG group was maintained. At T24, all FSFI parameters were significantly better in the VEL+Nd:YAG group.

**Table 6:** Changes in scores of FSFI. Table each date shows average ± standard deviation. *: Significant difference between the two groups. VEL: vaginal erbium SMOOTH mode laser treatment; VEL+Nd:YAG: VEL and neodymium-doped yttrium-aluminum-garnet laser combination treatment; FSFI: Female Sexual Function Index

	VEL+Nd:YAG group	VEL group	P-value*
Number of women	89	89	
T0			
FSFI	15.87 ± 4.69	15.57 ± 4.21	0.664
Desire	2.78 ± 0.85	2.64 ± 0.77	0.246
Arousal	2.44 ± 0.63	2.44 ± 0.63	0.501
Lubrication	2.58 ± 0.84	2.59 ± 0.77	0.889
Orgasm	2.41 ± 0.8	2.37 ± 0.72	0.571
Satisfaction	2.88 ± 1.09	2.74 ± 0.98	0.370
T1			
FSFI	20.89 ± 5.42	18.06 ± 5.86	0.001*
Desire	3.51 ± 1.01	3.03 ± 1.01	0.002*
Arousal	3.36 ± 0.87	2.85 ± 0.93	<0.001*
Lubrication	3.44 ± 0.91	3.02 ± 1.02	0.004*
Orgasm	3.26 ± 0.94	2.79 ± 1.00	0.001*
Satisfaction	3.72 ± 1.22	3.13 ± 1.20	<0.001*
T3			
FSFI	20.47 ± 5.97	15.88 ± 4.71	<0.001*
Desire	3.40 ± 1.01	2.66 ± 0.83	<0.001*
Arousal	3.31 ± 1.00	2.66 ± 0.83	<0.001*
Lubrication	3.37 ± 1.04	2.66 ± 0.85	<0.001*
Orgasm	3.20 ± 1.03	2.43 ± 0.81	<0.001*
Satisfaction	3.65 ± 1.27	2.76 ± 1.04	<0.001*

## Discussion

Our study evaluated the efficacy of the VEL and Nd:YAG laser combination treatment on GSM with dyspareunia and vulvodynia.

The main symptoms of GSM are a combination of urinary, sexual, and genital symptoms. Dyspareunia and vulvodynia are complex with a longstanding debate on where to target treatment. When this study was conceived, sexual pain was connected with vaginal dryness, and laser therapy was devised as a treatment for the vagina. In our supplement study, vaginal dryness in BCS was prominent in VHIS. This leads us to believe that the vagina is the target of treatment. Vaginal dryness improved with treatment in both the VEL and VEL+Nd:YAG groups.

However, a study by Goetsch et al. in 2014 reported that the cause of dyspareunia was the vulva, not the vagina, in women with BCS by performing swab tests on the vagina and labia [23]. In their swab test, para-hymen had the most pain, but no pain was noted in the vagina. This refutes the hypothesis that vaginal dryness causes dyspareunia. Our vulvodynia swab tests showed the same results as Goetsch et al. In particular, the most painful areas were at 3-4 o’clock and 8-9 o’clock of the para-hymen [23]. Only 1.28-2.8% reported pain in the vagina. Our results show that the VEL+Nd:YAG group, with the addition of vulvar laser treatment, is superior to the VEL group in both VAS and FSFI studies for dyspareunia.

A two-year long-term study on VEL for GSM with dyspareunia and vulvodynia was reported in 2018 [13] and on VEL for BCS in 2017 [13]. However, these were inadequate treatments for the vaginal vestibule and opening. More recently, in 2022, two innovative prospective pilot studies were reported using VEL+Nd:YAG laser for GSM [18] and erbium:YAG SMOOTH laser hyperstacking treatment for BCS [26]. The hyperstack mode uses a frequency of 1.6 Hz, a 7 mm spot, has a very low fluence of 1.5 J/cm, and releases 27 stacks for each spot. It was developed to achieve the same effect as the Nd:YAG laser.

Our study followed these two pilot studies [18,26]. It confirmed that treating the vaginal vestibule and vaginal opening with VEL+Nd:YAG reduces superficial vulvar pain more efficiently, to a greater extent, and for a longer period than VEL alone. However, the two previous studies [18,26] were prospective, whereas our retrospective study evaluated the population using PS analysis. In the study conducted on BCS [26], the study population was aged 59.53 ± 7.64 years, and YSM was 9.00 ± 5.47 years in the hyperstack group, and 59.11 ± 6.48 and 10.52 ± 6.08 years in the VEL group, respectively. In the study on GSM [26], the mean age of the study population was 62.3 ± 3.77 years, and YSM was 12.6 ± 5.55 years in the VEL+Nd:YAG group, and 60.00 ± 2.98 and 9.80 ± 4.44 years in the VEL group, respectively. Our study findings were consistent with the reported studies. In the study conducted on BCS [18], VAS was 9.74 ± 0.67 in the hyperstack group and 9.59 ± 0.66 in the VEL group, suggesting that pain levels were similar to those in our study population.

In a study investigating GSM with Nd:YAG [18], when the effect of VEL+Nd:YAG treatment was compared to that of VEL treatment alone, the Δ_T0/T3_ values ​​were -6.27 ± 1.71 (VEL+Nd:YAG group) and -3.13 ± 1.25 (VEL group). Our study confirmed this difference in treatment effects between the two groups. In addition, our retrospective analysis suggests that the more pronounced effects exerted by VEL+Nd:YAG are longer lasting and show a persistent, significant reduction in superficial vulvar pain two years after the final treatment, as opposed to what was observed in the VEL group.

Two pilot studies [18,26] reported only minor complications, consistent with our study. This study demonstrated that the efficacy of the laser treatment was not dependent on age or YSM, suggesting similar efficacy in all PMW with superficial vulvar pain. Large-scale studies have shown that VEL treatment has an exceptionally low-risk profile and a very low incidence of temporary and mild-to-moderate adverse events [20]. It is worth noting that VEL+Nd:YAG treatment was associated with fewer long-term complications in our study.

Combining the two pilot studies [18,26] and our study, we speculate that additional treatment for atrophy of the posterior commissure may help treat superficial vulvar pain and show long-term efficacy. Atrophic changes in the vestibular mucosa have been observed in many GSM women [3]. In a study that measured vestibular mucosal thickness, there were no significant differences between VBD (mean ± SD = 1,092.5 ± 226.1 μm) and the GSM groups (1,059.7 ± 221.5 μm); however, vestibular mucosa was significantly thinner in both the groups than that in the control group (1,310.6 ± 250.0 μm) [5]. Correlation analysis between reduced vestibular mucosal thickness and symptom intensity (burning/pain and vulvar pain) in the VBD and GSM groups has shown a significant correlation [27]. The Nd:YAG laser is reported to relieve vulvar tissue inflammation and regenerate normal tissue [27,28]. A dermatology study showed that the long-pulsed Nd:YAG laser has been an effective and safe technique for skin rejuvenation [29]. The same article reported that the use of vitamin C sonophoresis along with the Nd:YAG laser may reduce the incidence of post-inflammatory hyperpigmentation, which is a major adverse effect after Nd:YAG laser treatment [29].

Our study had some limitations. Owing to our research design, we could not evaluate the effects of local and systemic therapy by combining VEL+Nd:YAG treatment with hormonal or non-hormonal therapy or stratify laser effects by former treatment, severity, and/or symptom continuation. There was no group in which only the vulva was treated with laser. Erel et al. reported on only the vulva using Er:YAG laser treatment for vulvodynia, and it can be said that this arm should be comparatively studied [30]. Further long-term, well-designed, controlled studies are needed to investigate the effects of two complementary laser VEL+Nd:YAG wavelength treatments compared with VEL and other treatments for superficial vulvar pain and dyspareunia in women with GSM. Application to other patient populations may be constrained because it only included sexually active BCS with GSM superficial vulvar pain.

## Conclusions

Our study showed that VEL and Nd:YAG combination treatment is effective as a novel EBD treatment for GSM with superficial vulvar pain and dyspareunia in BCS. Over two years, the combination treatment was more effective than VEL alone. Combination therapy had no significant adverse events.

The results of the vulvodynia swab test, VHIS, and FSFI suggest that the vulva and the vagina are important therapeutic targets for pain in BCS with GSM. The importance of treating the vulvar area in GSM has been emphasized.
